# Initial damage and failure load of zirconia-ceramic and metal-ceramic posterior cantilever fixed partial dentures

**DOI:** 10.1007/s00784-024-05501-z

**Published:** 2024-01-15

**Authors:** Wolfgang Bömicke, Philipp Boisserée, Peter Rammelsberg, Stefan Rues

**Affiliations:** 1 Department of Prosthetic Dentistry, University Hospital Heidelberg, University of Heidelberg, Im Neuenheimer Feld 400, 69120 Heidelberg, Germany; 2 Hansezahn Zahnarztpraxen, Rodigallee 250, 22043 Hamburg, Germany

**Keywords:** Zirconium dioxide, Fracture load, Load-bearing capacity, Implant fixed dental prosthesis, Monolithic zirconia, Translucent zirconia

## Abstract

**Objectives:**

The aim of this study was to compare failure load and initial damage in monolithic, partially veneered, and completely veneered (translucent) zirconia cantilevered fixed partial dentures (CFPDs), as well as completely veneered metal-ceramic CFPDs under different support and loading configurations.

**Materials and methods:**

Eight test groups with anatomically congruent CFPDs (*n* = 8/group) were fabricated, differing in CFPD material/support structure/loading direction (load applied via steel ball (Ø 6 mm) 3 mm from the distal end of the pontic for axial loading with a 2-point contact on the inner cusp ridges of the buccal and oral cusps and 1.3 mm below the oral cusp tip for 30° oblique loading): (1) monolithic zirconia/CoCr abutment teeth/axial, (2) monolithic zirconia/CoCr abutment teeth/oblique, (3) partially veneered zirconia/CoCr abutment teeth/axial, (4) partially veneered zirconia/CoCr abutment teeth/oblique, (5) completely veneered zirconia/CoCr abutment teeth/axial, (6) completely veneered CoCr/CoCr abutment teeth/axial (control group), (7) partially veneered zirconia/implants/axial, and (8) partially veneered zirconia/natural teeth/axial. Restorations were artificially aged before failure testing. Statistical analysis was conducted using one-way ANOVA and Tukey post hoc tests.

**Results:**

Mean failure loads ranged from 392 N (group 8) to 1181 N (group 1). Axially loaded monolithic zirconia CFPDs (group 1) and controls (group 6) showed significantly higher failure loads. Oblique loading significantly reduced failure loads for monolithic zirconia CFPDs (group 2). Initial damage was observed in all groups except monolithic zirconia groups, and fractography revealed design flaws (sharp edges at the occlusal boundary of the veneering window) in partially veneered zirconia CFPDs.

**Conclusions:**

Monolithic zirconia CFPDs might be a viable alternative to completely veneered CoCr CFPDs in terms of fracture load. However, oblique loading of monolithic zirconia CFPDs should be avoided in clinical scenarios. Design improvements are required for partially veneered zirconia CFPDs to enhance their load-bearing capacity.

**Clinical relevance:**

Monolithic zirconia may represent a viable all-ceramic alternative to the established metal-ceramic option for CFPD fabrication. However, in daily clinical practice, careful occlusal adjustment and regular monitoring should ensure that oblique loading of the cantilever is avoided.

## Introduction

The cantilever fixed partial denture (CFPD) has been defined as a fixed restoration that has one or more abutments at one end, with the other end unsupported [1]. In principle, CFPDs can replace any tooth in the dental arch, but are considered particularly useful for avoiding a removable partial denture in patients with distal edentulism [2] or for establishing the minimum acceptable number of occlusal units in the shortened dental arch concept [3]. Favorable outcomes can be expected for CFPDs that have at least two abutment teeth and do not replace more than one tooth [3–5]. The survival rate of CFPDs with two abutment teeth that were vital at the time of cementation and had at least two-thirds residual alveolar bone was comparable to that of conventional endabutment FPDs [6].

Even in the age of implants, CFPDs can be useful because there are still patients who cannot afford implant treatment or for whom implant treatment is not an option or is too complicated due to insufficient bone or other reasons [7]. In addition, restoration of edentulous ridges adjacent to implants using cantilevers may be considered for esthetic or economic reasons or to avoid greater augmentation effort without negatively affecting peri-implant health or increasing the risk of mechanical complications [8–11]. A recent review specified that the use of CFPDs on implants did not have a negative impact on prosthesis survival or success or marginal bone loss [12]. The unique biomechanics, with eccentric forces acting on both restorations and abutments, place high demands on the design of CFPDs and the properties of the materials used in their fabrication. This involves ensuring that the CFPDs are durable over the long term under the forces acting in the oral cavity. Here, metal-ceramic CFPDs represent the therapeutic standard [11, 13, 14].

Biological, economic, and aesthetic considerations have led to the increasing use of zirconia in place of traditional metal ceramics [15]. Early clinical experience with mostly completely veneered zirconia CFPDs on teeth [13, 16] and implants [14, 17–19] indicates that the success of restorations on teeth is mainly limited by chipping [13, 16], whereas implant-supported CFPDs also experience framework fractures [14, 18]. At the same time, in vitro studies cast doubt on whether the load-bearing capacity of completely veneered zirconia CFPDs is sufficient for molar replacement, despite numerous design modifications involving reinforced zirconia frameworks [20–23].

Recent developments have focused on monolithic full-contour zirconia restorations [24], also for CFPDs [25]. The monolithic design has two major advantages for CFPDs. Firstly, the veneer is no longer a potential site of initial damage under load [26, 27], which should reduce the risk of chipping [28]. Secondly, the space that would be taken up proportionally by the much weaker veneering ceramic can be used entirely for the high-strength zirconia, which should maximize the load-bearing capacity of ceramic CFPDs [29]. The acceptance of such monolithic restorations depends not only on the strength of the material but also on esthetic criteria. For this reason, early attempts were made to improve the translucency of the available high-strength 3-mol yttria-stabilized zirconia polycrystal (3Y-TZP) materials. This was achieved with the 2nd generation zirconia by reducing the amount of alumina in 3Y-TZP and by increasing the sintering temperature [30]. The result was acceptable esthetics for the posterior region while maintaining maximum flexural strength. In subsequent generations, the yttria content was increased to 4 mol% (4 mol% partially stabilized zirconia, 4Y-PSZ) and 5 mol% (5Y-PSZ) to further improve translucency [30]. The increased yttria content improves translucency by increasing the mean grain size and proportionally stabilizing the cubic phase, which has an isotropic crystal structure and thus more uniform light scattering compared to the tetragonal phase. However, because the cubic phase does not have the property of transformation toughening, the flexural strength values of these zirconia materials is lower [30]. Nowadays, there are also multilayer materials combining several types of zirconia in one milling disk (translucent layers located in the area of the incisal edges/cusp tips) and thus exhibiting a strength/translucency gradient [31]. However, for monolithic zirconia single crowns and FPDs made of 2nd generation zirconia, it has been shown that a facial veneer in the anterior and premolar region can achieve high patient satisfaction with regard to restoration esthetics without measurably increasing the restorations’ risk of technical complications [28, 32]. Such a design variant would also be interesting for zirconia CFPDs, especially since it can be assumed that the critical stresses during loading of the pontic occur mainly on the upper site of the restorations [5, 25, 33].

Accordingly, the aim of this in vitro study was to compare the initial damage and failure loads after artificial aging and under different loading conditions (axial/oblique) of monolithic, partially (facially), or completely veneered posterior 2nd generation zirconia CFPDs attached to either cobalt–chromium (CoCr) or natural abutment teeth or implants. Axially loaded completely veneered CoCr CFPDs attached to CoCr abutment teeth served as the control. The null hypothesis was that there would be no differences between the different CFPD design, loading, and abutment groups.

## Material and methods

**Fig. 1:**
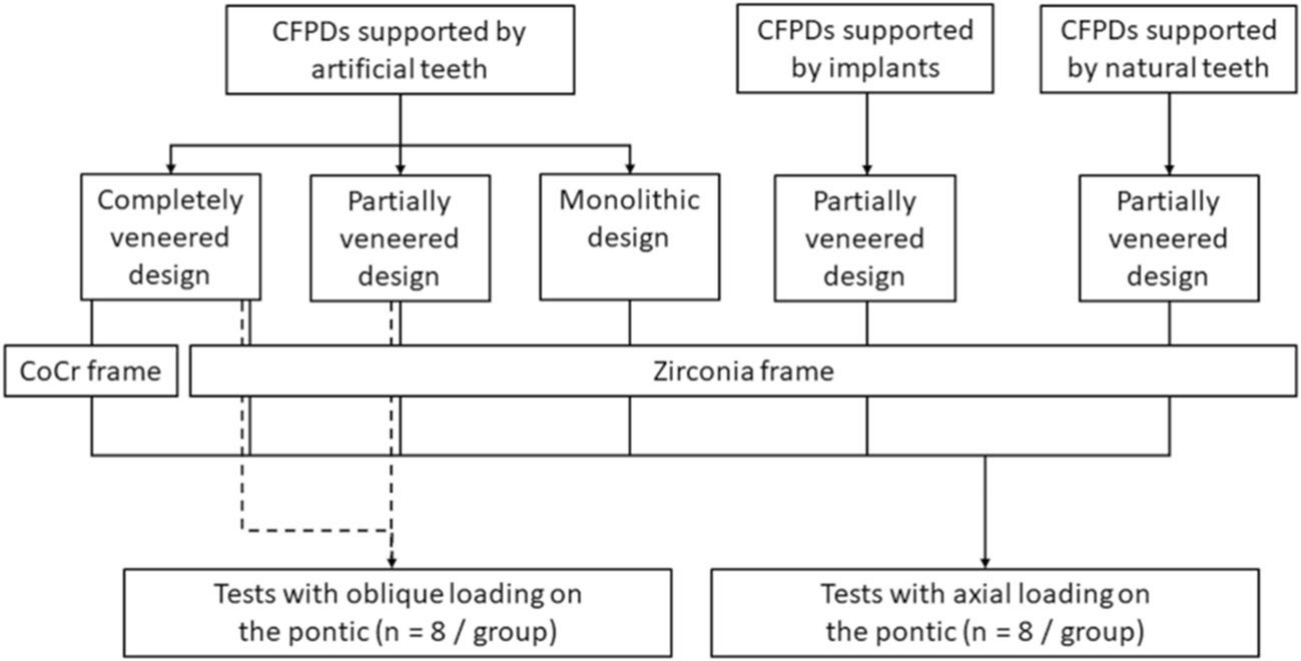
Test group flow chart

**Fig. 2:**
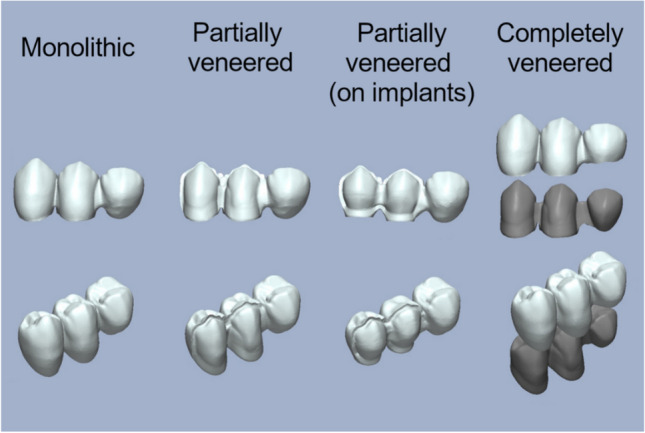
Overview of CFPD three-dimensional (3D) designs underlying test groups

CFPDs were designed to replace a lower first molar by means of a distal cantilever in the shape of a premolar (mesio-distal dimension: 8 mm) retained by 2 splinted complete crowns attached to the first and second premolar. A mandibular typodont (type ANA-4, Frasaco, Tettnang, Germany) was used as the anatomical basis for the experiments. The prepared typodont teeth (see below) were then replicated in CoCr (Remanium GM800, Dentaurum, Ispringen, Germany) and served as abutments in the test models. In addition to testing CFPDs on the CoCr tooth replicas, CFPDs attached to implants (tissue level implants, SP, RN, SLA, Roxolid, diameter 4.1 mm, length 10 mm, Straumann, Basel, Switzerland) or natural teeth were also tested. Three different designs (monolithic, partially veneered, completely veneered) for zirconia CFPDs were tested with abutment tooth replicas. Zirconia CFPDs supported by implants or natural teeth were only investigated with a partially veneered design. As a control, the completely veneered design was also tested with a CoCr framework. All groups were exposed to axial loading on the pontic during aging and fracture tests. Additional tests with oblique loading (30° tilt) on the pontic were conducted for monolithic and partially veneered CFPDs supported by abutment tooth replicas (Fig. 1). Based on a previous study of similar restorations [22], a sample size of *n* = 8 per group was considered adequate to detect statistically significant differences with adequate power. The CFPDs in the different test groups were designed to be congruent with respect to their external geometry (Fig. 2).

### Design of CFPDs based on the prepared typodont teeth

The typodont abutment teeth were prepared with an axial and occlusal reduction of 1.5 mm, a 0.5-mm deep chamfer finishing line, rounded edges, and a total occlusal convergence of the axial walls of 6° using a paralleling device. A type-IV gypsum master cast (GC Fujirock-EP, GC Europe, Leuven, Belgium) of the situation was digitized by use of a laboratory scanner (D800, 3Shape, Copenhagen, Denmark).

First, a completely veneered CFPD was designed (Dental Designer, 3Shape) using the situation with unprepared abutment teeth as a wax-up scan. The framework had a minimum layer thickness of 0.8 mm and supported the veneer anatomically (Fig. 2). The connectors were set to minimum cross-sections of 9 mm^2^ between the abutments and 12 mm^2^ for the cantilever (Table 1). Second, by combining framework and veneer using 3D manipulation software (Geomagic Design X, 3D Systems, Moerfelden-Walldorf, Germany), a monolithic CFPD with identical outer geometry was created (Fig. 2). Third, the monolithic design was reduced on the buccal side of the crown retainers by 0.7 mm to provide space for a partial veneering (Fig. 2). Therefore, the area of the veneering window was marked in the software (Geomagic Design X, 3D Systems) and reduced by the appropriate amount. At the boundaries, the transition areas were given a profile with a radius (0.4 mm radius → 0.5 mm radius in the scaled situation during milling) corresponding to the smallest milling tool (1 mm in diameter) used on this surface.

**Table 1:** Connector cross-sections of CFPD design variants

CFPD design	Connector cross-section between abutments (mm^2^)	Connector cross-section at cantilever (mm^2^)
Monolithic	18.8	21.1
Partially veneered (tooth-supported)	13.8	20.6
Partially veneered (implant-supported)	12.4	16.7
Completely veneered	9.6	12

### Design of partially veneered CFPDs supported by implants

The implants were used with standard abutments (RN synOcta Cementable Abutment, height 5.5 mm; Straumann, fixation torque 35 Ncm). The digitized geometry of an implant with standard abutment was placed at each abutment tooth position of the partially veneered design described above. The implant axes were oriented vertically, and their spatial position was chosen such that the implant neck center was identical to the center of the respective abutment tooth margin line in horizontal and vertical direction. Inner surfaces of crowns designed on the implants with abutments were complemented by the existing outer CFPD geometry (Fig. 2).

### Design of partially veneered CFPD supported by natural teeth

Natural premolar teeth were implemented in a gypsum model such that they resembled the typodont situation as closely as possible, i.e., parallel tooth axes and a tooth center distance of 7.5 mm. After preparation under microscopic control, tooth dimensions did not deviate from those of the typodont teeth situation by more than 0.5 mm in any spatial direction. Natural teeth in the study were allowed to have small defects that did not affect the pulp system. After caries excavation and prior to preparation, such defects were filled with a composite resin (Rebilda DC, VOCO, Cuxhaven, Germany) using a total-etch adhesive technique (Primer and Adhesive, Optibond FL, Kerr, Kloten, Switzerland). For the use of natural teeth, a positive ethics vote was available (S-034/2010), and tooth donors signed an informed consent form. Until their use, the teeth were stored in 1% chloramine-T solution.

For CFPDs on natural abutment teeth, the veneer of the completely veneered design (based on the typodont teeth) was milled from wax and positioned as congruent as possible over the prepared natural teeth using a paralleling device. Missing contours up to the preparation margins were completed with wax. This situation was digitized and used as a wax-up scan for the CFPD design. Partial reduction of the crown retainers on the buccal side by 0.7 mm was done individually at the end.

### Standardization of loading site

**Fig. 3:**
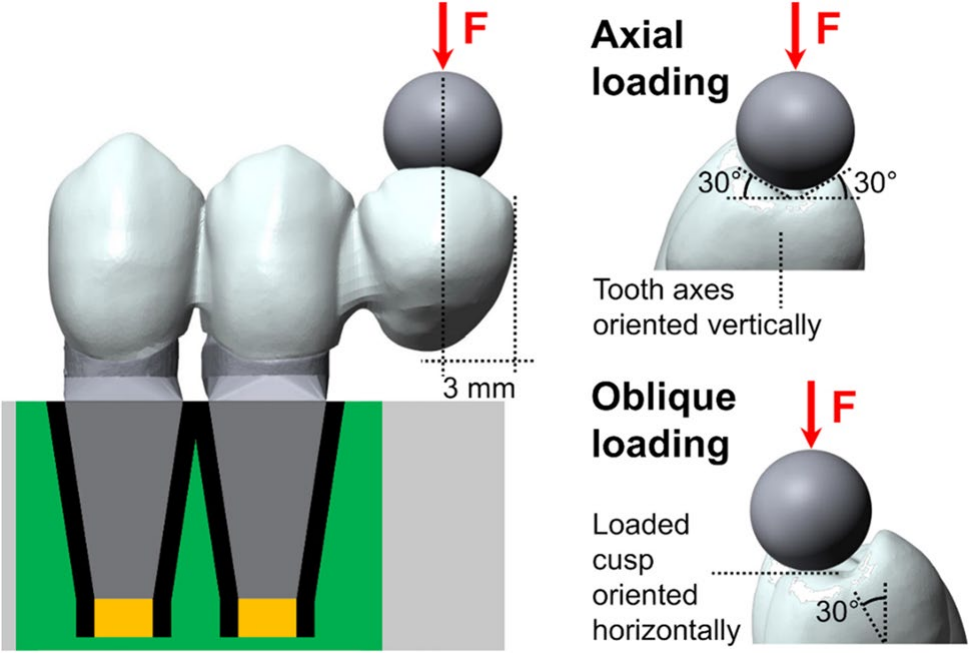
Graphic illustration of modified occlusal surface on cantilever to provide standardized loading site for axial and oblique loading, and test model with abutment teeth resiliently embedded in acrylic resin (green) using heat-shrink tubing (black) and polyvinylsiloxane (yellow)

The cantilever pontic was modified such that the inner ridges of the cusps were planar and featured angles of ±30° to the horizontal direction and the mesial-distal axis as rotational axis (Fig. 3). This enabled a standardized loading of all CFPDs.

### CFPD fabrication

Zirconia frameworks were centrally milled (CNC 500 milling unit, 3M Oral Care, Seefeld, Germany) from translucent (2nd generation) 3Y-TZP (Lava Plus Multi L, 3M Oral Care), monochromatically dyed (A4) by immersion for 2 min in an appropriate dyeing solution (Lava Plus Dyeing Liquid, 3M Oral Care), dried for 2 h at room temperature, and sintered at 1450 °C (Lava Furnace 200, 3M Oral Care). Monolithic zirconia frameworks subsequently received two glaze firings (VITA AKZENT GLAZE SPRAY, VITA Zahnfabrik, Bad Säckingen, Germany). For completely veneered zirconia CFPDs, the zirconia frameworks were overpressed (IPS e.max ZirPress HT A4, Ivoclar Vivadent, Schaan, Lichtenstein) according to the manufacturer’s instructions for the veneering ceramic. The 3D-designed veneer was milled in wax (LAWAX, 3M Oral Care) to serve as a space holder during the overpressing procedure. After liner firing (ZirLiner Liquid Build Up Allround and Zir Liner Clear, Ivoclar Vivadent), the wax-milled veneer was positioned on the zirconia framework and manually supplemented with wax at the basal/cervical aspects using a putty silicone negative of the monolithic CFPD as a mold. Two glaze firings (IPS e.max Ceram Glaze Paste) completed the fabrication process. Partially veneered zirconia CFPDs were finalized using the layering technique (VITA VM 9, VITA Zahnfabrik) and two subsequent glaze firings (AKZENT GLAZE SPRAY, VITA Zahnfabrik). Completely veneered metal-ceramic CFPDs served as the control. Therefore, the anatomically reduced framework of the respective all-ceramic group was milled from wax and cast with CoCr alloy (Remanium Star, Dentaurum). The veneering was carried out analogously to the completely veneered zirconia CFPDs using leucite-containing pressable ceramics (IPS InLine PoM A4, Ivoclar Vivadent) and finalized with two glaze firings (IPS Ivocolor Glaze Paste, Ivoclar Vivadent). All CFPDs were checked for marginal and internal fit and adjusted manually if necessary.

### Model fabrication and cementation

Roots of CoCr abutment tooth replicas as well as natural teeth were coated with a heat-shrink tubing (HIS-A 12/4-PO-X-BK, HellermannTyton, Tornesch, Germany) to achieve realistic tooth mobility during the tests. The shrink tubing was cut off 2 mm below the apical end of the root and the opening filled with polyvinylsiloxane (Flexitime Correct Flow, Kulzer, Hanau, Germany) (Fig. 3). Using molds resembling the negative shape of the occlusal surface and a paralleling device, abutment tooth replicas, implants, or natural teeth provisionally fixed in the respective CFPD were embedded in acrylic resin (Technovit 4071, Kulzer) in a metal specimen holder in the planned position and orientation (Fig. 3).

For CFPD cementation, the intaglio surfaces of the zirconia crown retainers were alumina-particle abraded at 0.1 MPa pressure (Alustral 50 μm, Omnident Dental-Handelsgesellschaft, Rodgau Nieder-Roden, Germany). CoCr crown retainers and CoCr abutment teeth as well as implant abutments were alumina-particle abraded with 0.2 MPa (Alustral 50 μm). Subsequently, CFPDs, CoCr abutment teeth, and implants were steam cleaned and thoroughly dried. Natural abutment teeth were cleaned with polishing paste (Zircate, Dentsply Sirona, Bensheim, Germany), rinsed with water, and lightly dried with oil-free air. Cementation was performed in a universal testing machine (Z005, Zwick/Roell, Ulm, Germany) with self-adhesive resin cement (RelyX-Unicem Automix 2 A3, 3M Oral Care) at 400 N applied for 180 s centrally between the abutment teeth. After storage for 24 h at 100% humidity and 37 °C in an incubator (Heraeus Functionline Heating Oven, Thermo Fisher Scientific, Waltham, MA, USA), CFPDs were examined under a stereo light microscope (Stemi SR, Zeiss Microscopy, Oberkochen, Germany; 8× magnification) for damage during cementation such as fractures, cracks, or chipping.

### Artificial aging and failure testing

All CFPDs were artificially aged using 10,000 thermocycles (bath temperatures 6.5 °C and 60 °C, Thermocycler TC 1, SD Mechatronik, Feldkirchen-Westerham, Germany) and 1.2 million chewing cycles (CS-4.8, SD Mechatronik) with a force magnitude of 108 N. During chewing simulation, samples were immersed in deionized water and a steel ball (Ø 6 mm) served as antagonist. For each group, loading conditions during aging resembled those used later on during the fracture tests. After artificial aging, CFPDs were inspected again for possible damage such as fractures, cracks, chipping, or decementation at up to 200× magnification (Stemi SR, Zeiss Microscopy).

Failure testing was performed in a universal testing device (Z005, Zwick/Roell) at a feed rate of 0.5 mm/min. Forces were applied with a steel ball (Ø 6 mm) 3 mm from the distal end of the pontic (Fig. 3). With axial loading, the test force was applied via contact on both cusps (Fig. 3). For oblique loading, samples were fixated with 30° tilt such that the loaded cusp was oriented horizontally (Fig. 3), and the force application point was 1.3 mm below the cusp tip. The end of the failure test was defined as when the test force decreased to less than 30% of the previous maximum value or damage equivalent to clinical failure occurred.

**Fig. 4:**
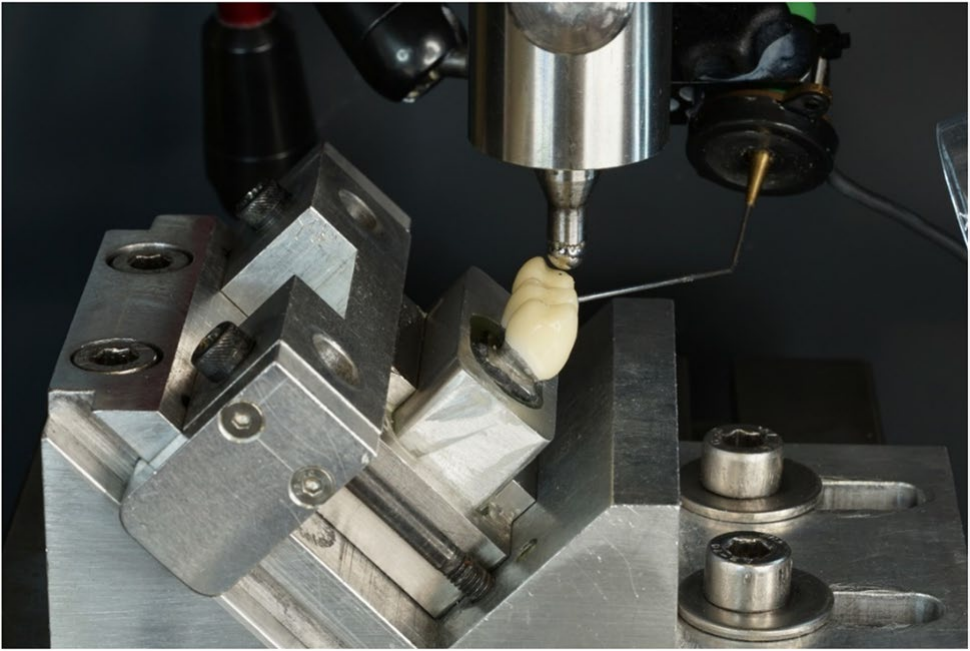
Completely veneered zirconia CFPD in universal testing machine prepared for oblique loading with contact microphone attached

During the failure tests, body-borne sound signals were recorded (20 kHz sampling rate) to help identify damage prior to CFPD failure (Fig. 4). A damage event was given for an interim drop in test force coinciding with a high sound signal exceeding 75% of the maximum magnitude recorded during the complete test. Forces at failure and the first damage event (initial damage) were recorded. If no pre-failure event occurred, initial CDFP damage coincided with CFPD failure. In case of initial damage or failure during artificial aging, a force of 108 N (force magnitude during chewing simulation) was associated with initial damage and/or failure.

### Failure modes and fractography

All tested CFPDs were examined by light microscopy and classified according to their failure modes. Representative specimens were fractographically examined by light (Stemi SR, Zeiss Microscopy) and scanning electron microscopy (SEM) using a field emission scanning electron microscope (Auriga 40, Carl Zeiss Microscopy; acceleration voltage: 1.5 kV, working distance: 3–6 mm) to identify fracture origin and crack propagation and thus get information about possible causes for the respective fracture. The fractographic examination was performed by the manufacturer of the ceramic framework material (3M Oral Care).

### Statistical evaluation

Test forces at failure and at initial damage were analyzed separately using one-way analysis of variance (ANOVA), and Tukey honest significant difference post hoc tests were used for the pairwise comparisons (2-sided *α* = 0.05).

## Results

**Fig. 5:**
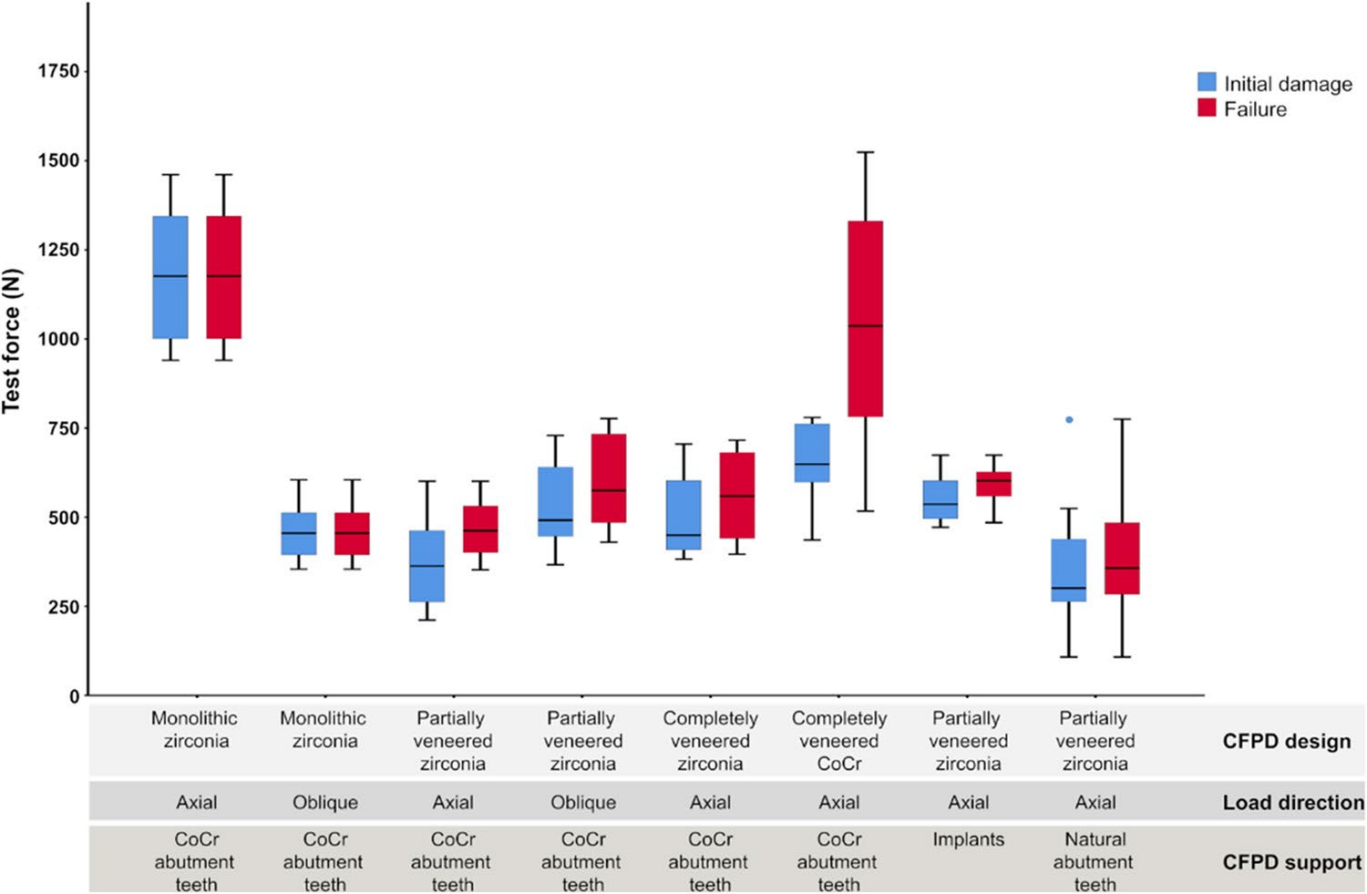
Whisker and box plots of test forces at initial damage and failure of CFPDs in test groups (*n* = 8 per test group)

Test forces corresponding with failure and initial damage are listed in Table 2 and shown in Fig. 5.

**Table 2:** Test forces at initial damage and failure of CFPDs in test groups (*n* = 8 per test group)

Test group (specifications)			Initial damage (*N*)			Failure (*N*)		
CFPD design	Load direction	CFPD support	Mean (SD)	Min	Max	Mean (SD)	Min	Max
Monolithic zirconia	Axial	CoCr abutment teeth	1181^a^ (199)	940	1461	1181^A^ (199)	940	1461
Monolithic zirconia	Oblique	CoCr abutment teeth	460^bc^ (83)	354	605	460^B^ (83)	354	605
Partially veneered zirconia	Axial	CoCr abutment teeth	373^c^ (134)	211	601	468^B^ (85)	352	601
Partially veneered zirconia	Oblique	CoCr abutment teeth	532^bc^ (132)	367	729	599^B^ (134)	430	777
Completely veneered zirconia	Axial	CoCr abutment teeth	501^bc^ (120)	382	705	559^B^ (129)	396	716
Completely veneered CoCr	Axial	CoCr abutment teeth	654^b^ (116)	436	780	1042^A^ (350)	517	1524
Partially veneered zirconia	Axial	Implants	552^bc^ (70)	472	674	592^B^ (58)	485	674
Partially veneered zirconia	Axial	Natural abutment teeth	361^c^ (203)	108	775	392^B^ (201)	108	775

### Failure load

Mean test forces at failure ranged between 392 N for axially loaded partially veneered zirconia CFPDs on natural teeth and 1181 N for axially loaded monolithic zirconia CFPDs on CoCr abutment teeth. The mean failure load of the axially loaded monolithic zirconia CFPDs was not statistically significantly different (*p* = 0.777) from that of the control group, which had a mean failure load of 1042 N. These two groups also had significantly higher mean failure loads than all other groups in the test (*p* < 0.001). Compared to axial loading, oblique force application led to statistically significantly (*p* < 0.001) lower fracture forces for monolithic zirconia CFPDs (460 N) and a slightly higher (no significant effect, *p* = 0.819) mean failure load for partially veneered zirconia CFPDs (599 N compared to 468 N).

**Fig. 6:**
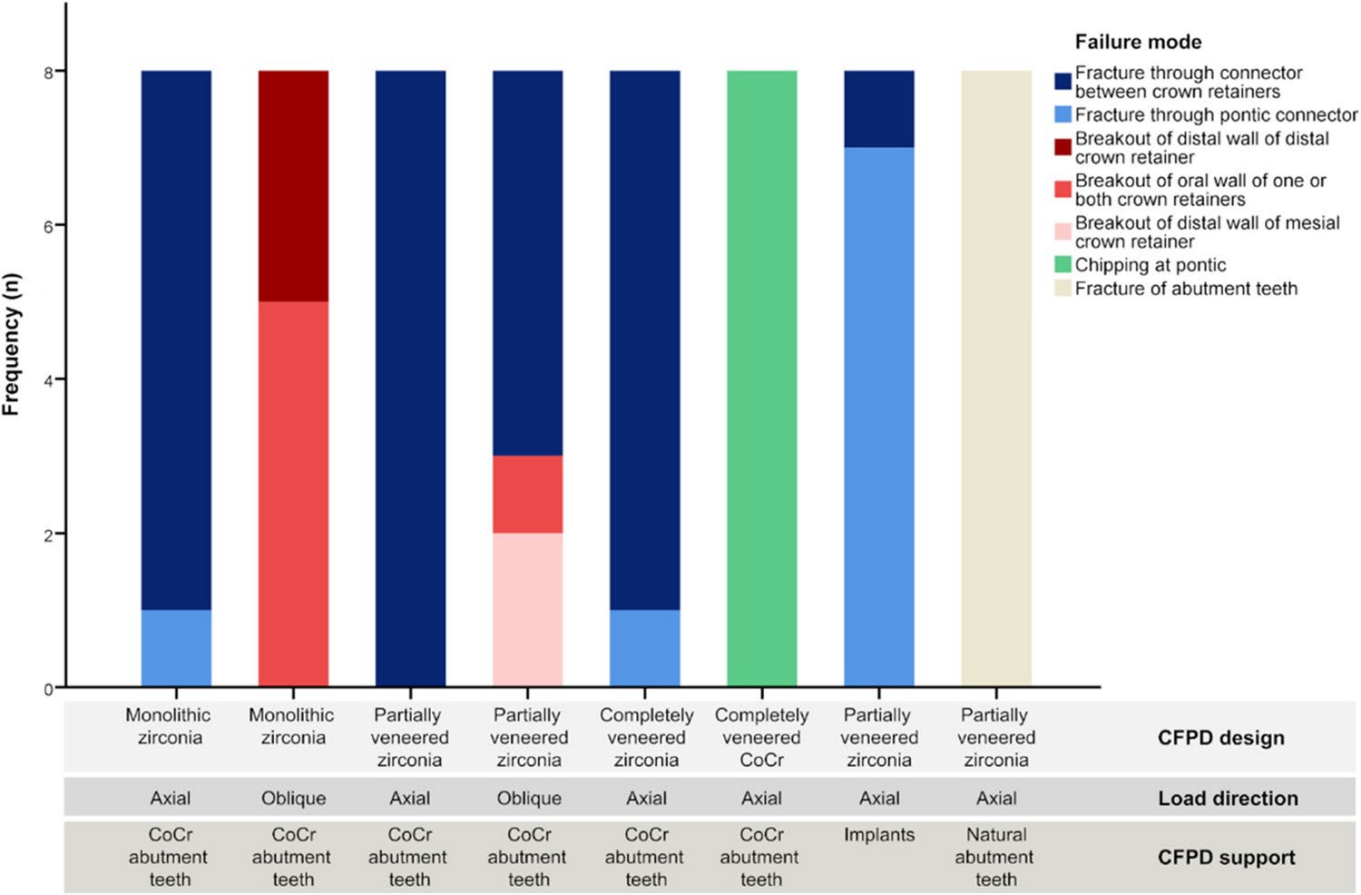
Frequencies of observed failure modes of CFPDs in test groups

The failures of all tested CFPD could be classified with eight different fracture modes (Fig. 6). The most common fracture was through the connector between the two crown retainers. Half of the test groups exclusively or predominantly showed this failure mode. Axially loaded monolithic or partially veneered groups on CoCr abutment teeth were particularly affected. For obliquely loaded CFPDs, however, a shift of the failure pattern towards a breakout of the retainer walls was observed. Also different were implant-supported CFPDs, completely veneered CFPDs (controls), and CFPDs on natural teeth: implant-supported CFPDs fractured through the pontic connector, controls failed exclusively due to excessive chipping of the veneering ceramics, and all natural tooth–supported CFPDs failed due to fractures of the abutment teeth.

### Initial damage

Initial damage before failure, i.e., crack formation or small chippings within the veneer, was registered for completely or partially veneered CFPDs. No initial damage events before the final fracture were observed in monolithic restorations. Mean test forces at initial damage reached from 361 N for axially loaded partially veneered zirconia CFPDs supported by natural abutment teeth to 1181 N for axially loaded monolithic zirconia CFPDs supported by CoCr abutment teeth. Axially loaded monolithic zirconia CFPDs differed significantly from control CFPDs (*p* < 0.001) with the second highest mean forces corresponding with initial damage (654 N), and from all other test groups (*p* < 0.001) showing initial damage below 500 N for most samples.

### SEM analysis and fractography

Analysis of SEM images of selected samples revealed three main types of detectable failure causes of the ceramic materials in this study: (1) process-related, (2) design-related, and (3) aging-related.

**Fig. 7:**
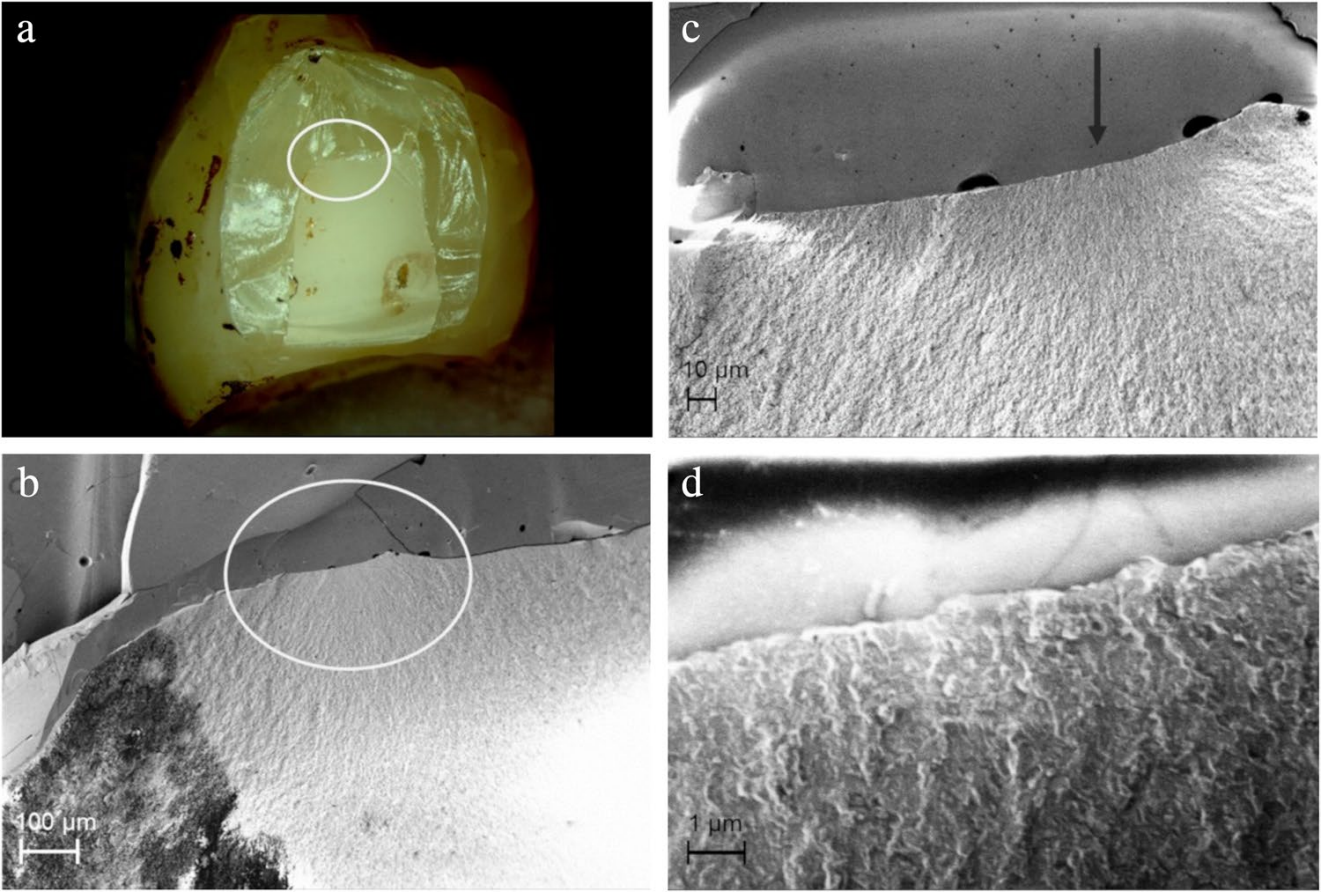
Axially loaded completely veneered zirconia CFPD with fracture through connector between crown retainers. **a** Circle marks origin of the fracture. **b**–**d** Fracture origin is within surface depression/milling path. Arrow points to wide area from which crack has developed. Defined fracture origin is not recognizable

**Fig. 8:**
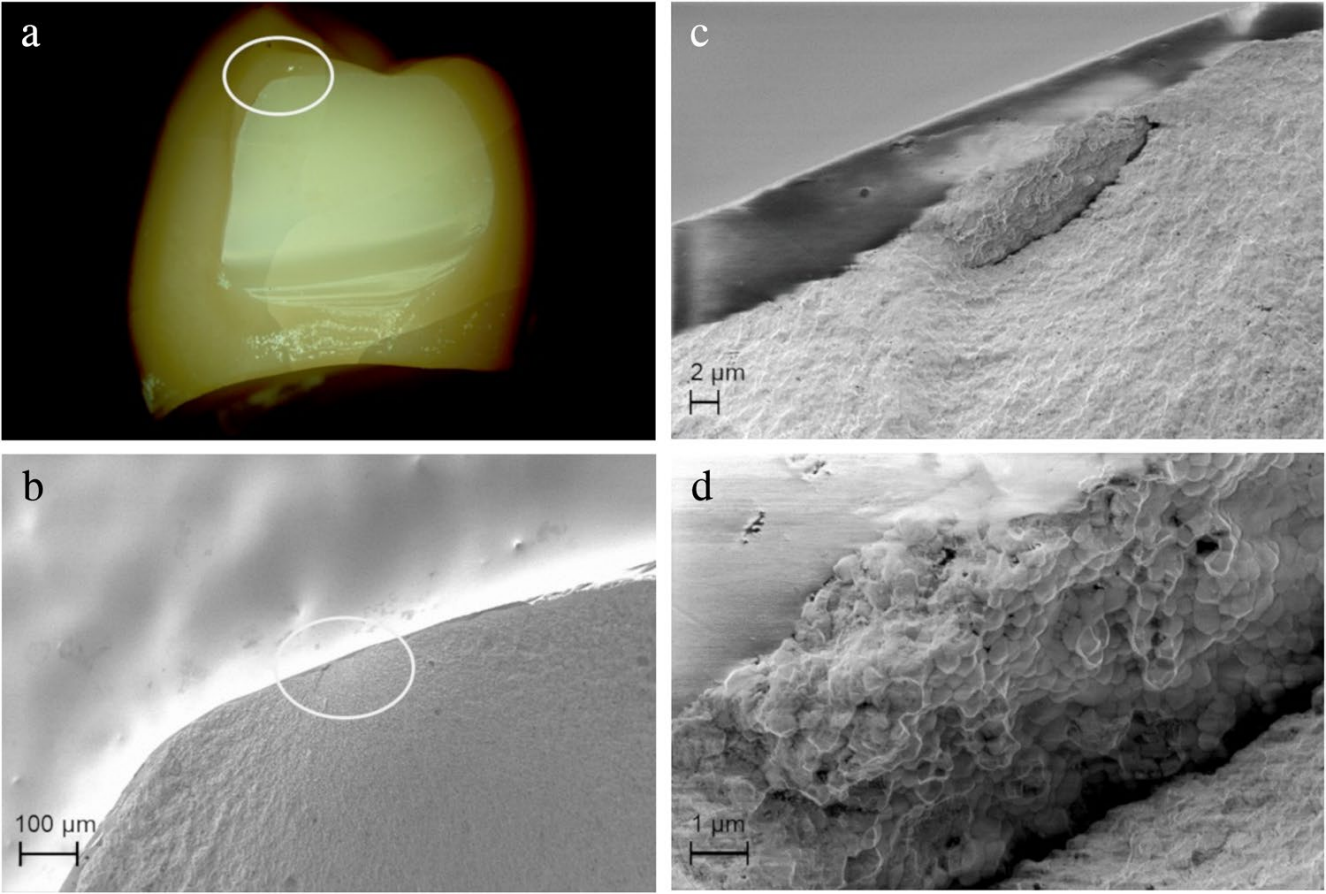
Axially loaded monolithic CFPD with fracture through connector between crown retainers. **a** Circle marks origin of fracture at top of connector. **b**–**d** With increasing magnification using SEM, chipping fracture caused by milling process becomes visible as microstructural starting point of restoration fracture

**Fig. 9:**
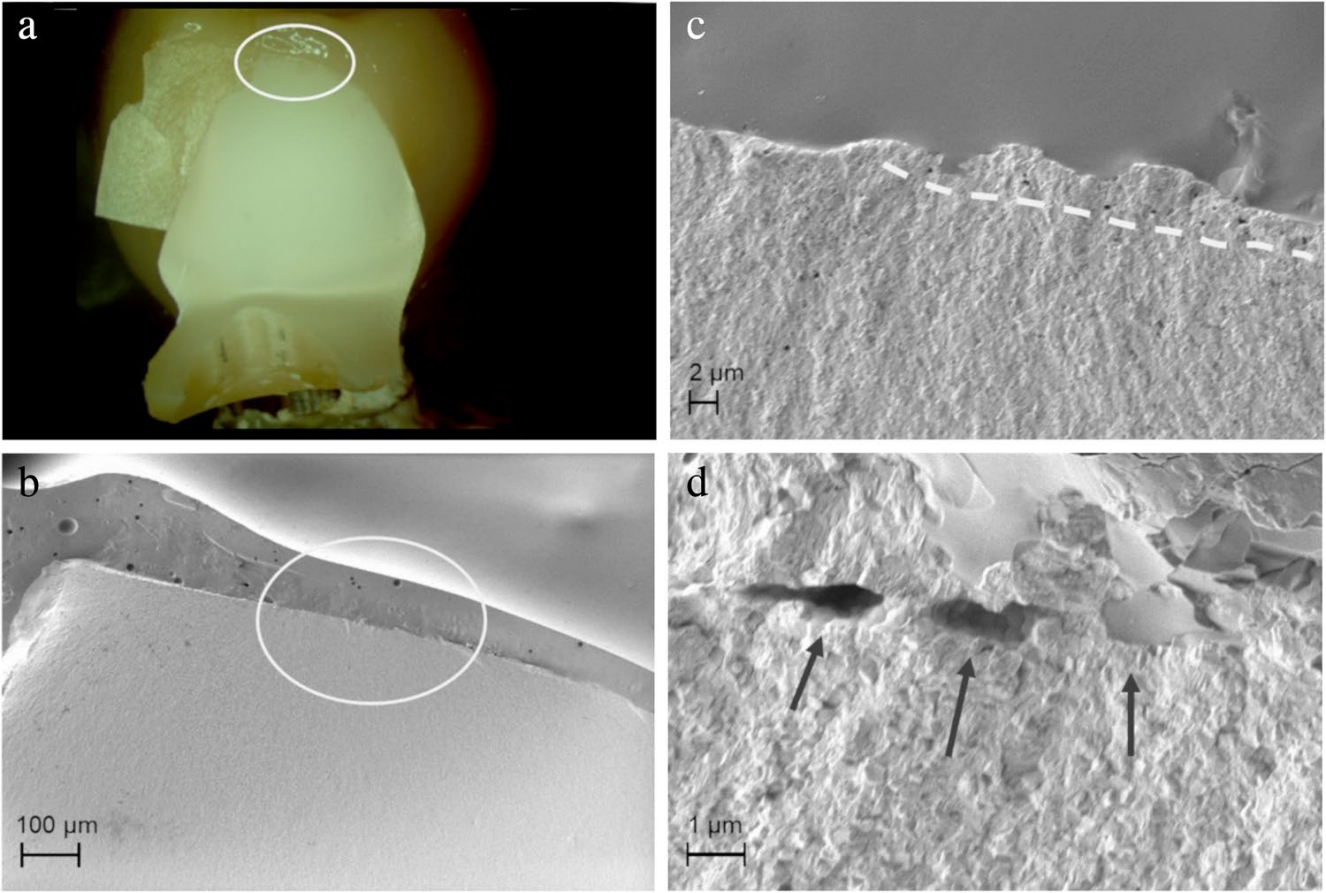
Fracture through pontic connector of axially loaded partially veneered implant-supported zirconia CFPD. **a** Circle marks fracture origin at top of connector. **b**–**d** Dashed line shows “pore line” visible under scanning electron microscope with pores partly infiltrated with glaze (arrows). This appears to be larger area of milling dust adhesion

In many samples, milling marks (Fig. 7) and small chippings (Fig. 8) on the zirconia framework surface caused by the milling process, and milling dust deposits as well as resulting superficial pore formation (Fig. 9) along the zirconia surface were found to be process-related flaws that could be identified as origins of fracture.

**Fig. 10:**
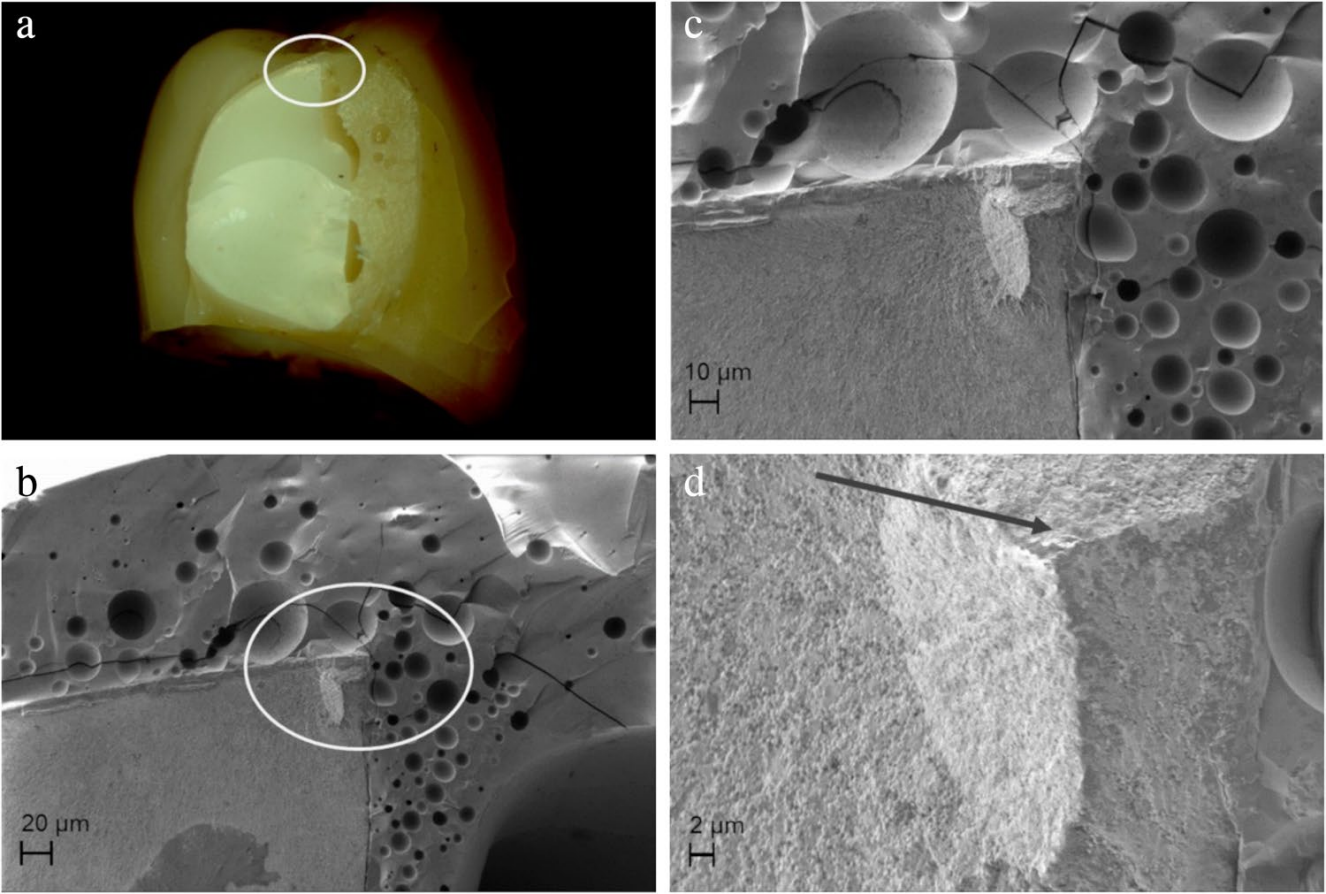
Failure through retainer connector of axially loaded partially veneered zirconia CFPD on CoCr abutment teeth originating from sharp edge at upper boundary of anatomical reduction for ceramic veneer. **a** Circle marks fracture origin at top of connector. **b**–**d** Sharp edge on zirconia framework is visible (circle) and fracture origin located there can be traced (arrow)

Design-related fractures originated from edges of the zirconia frameworks where stress concentrations could occur. Such “edge effects” were observed predominantly in partially veneered CFPDs in the area of the sharp-edged boundary of the anatomical reduction for the ceramic veneer (Fig. 10).

**Fig. 11:**
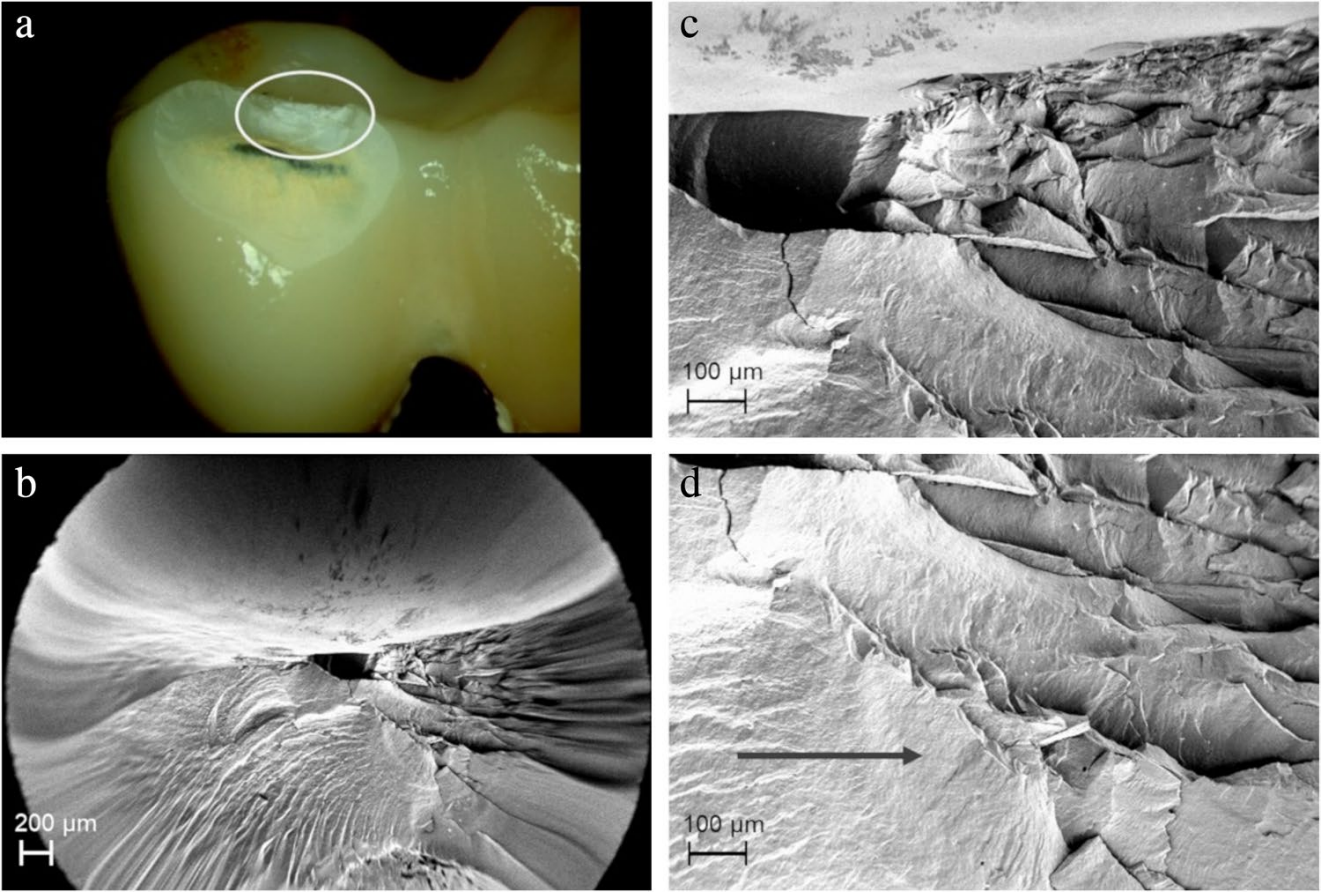
Completely veneered CoCr CFPD failing from extensive veneer chipping. **a** Circle marks fracture origin in area of load indenter. **b**–**d** Shapes around fracture area indicate failure pattern where crack propagation was gradual. Arrow indicates origin of crack. Fracture pattern is consistent with that seen in multiple “Hertzian compression”

Aging-related damage origins were detected for the control group (completely veneered CFPDs with CoCr frame) where veneer chipping started at the loading site in the area of pre-damage caused by “Hertzian compression” (Fig. 11).

## Discussion

This study tested the hypothesis that there would be no differences between the loads at failure and initial damage between three-unit CFPDs for the replacement of a premolar-size tooth as a function of restoration design (monolithic/partially veneered/completely veneered zirconia or completely veneered CoCr), loading (axial/oblique), and abutment type (CoCr teeth/natural teeth/implants). The hypothesis had to be partially rejected.

Highest failure loads for CFPDs were associated with axial loading and resiliently embedded CoCr abutment teeth and occurred for monolithic zirconia CFPDs and completely veneered restorations with a CoCr framework. All other test groups differing in loading/support conditions and design, showed significantly lower fracture resistance. This result was to be expected as the monolithic zirconia CFPDs had the largest dimension of the zirconia framework compared to the partially and completely veneered zirconia restorations, and it is known that the loading capacity of a ceramic restoration depends not only on the flexural strength but also on geometric parameters such as wall thickness [34]. For CFPDs with a CoCr framework, the metal only deformed plastically until large parts of the veneering got lost, thus leading to a high fracture resistance of the entire restoration [35]. For a dentist, however, an initial damage may already be a failure of a restoration. Restorations of all test groups but monolithic zirconia CFPDs experienced initial damage in the form of cracking in the veneering ceramic at loads about half of the respective failure loads. Knowledge of this initial damage allows the results to be better transferred to a clinical context where veneer chipping is a common complication of tooth- or implant-supported CFPDs [36, 37]. Even if initial damage (chipping, cracks) does not result in immediate clinical restoration failure, it can influence restoration failure over time [38].

Related to maximum achievable bite forces of 800 N and 600 N for young adult males and females [39] and chewing forces at about 40% of the maximum bite force [40], the results of the current study suggest that only monolithic zirconia CFPDs and veneered restorations with a CoCr framework will provide clinically acceptable failure loads. This finding is somewhat put into perspective when considering the forces that occur in an older age group, which corresponds more to a prosthetic patient population. Completely dentate patients with an average age of 70.2 years were found to exhibit mean maximum bite forces of 377 N in the first molar region [41]. Furthermore, there are indications that for eccentric forces on cantilevers self-inhibition mechanisms of the masticatory system may limit the maximally exerted force. Lundgren and Laurell reported that the maximum individual bite force was 150 N in patients treated with cross-arch FPDs with unilateral posterior cantilevers when the occlusal load was actively focused on the cantilever [42]. This may suggest that, during clinical function, CFPDs are not subjected to bite forces as high as those previously described. This assumption is indirectly supported by the fact that in clinical studies of tooth-supported zirconia CFPDs, no framework fractures have been observed [13, 16]. The situation is somewhat different for implants, where the feedback mechanisms described above are less likely to be effective. For example, over a period of 10 years, framework fracture was identified as the most frequent cause of failure for both CFPDs and end-abutment FPDs on implants in posterior dentitions [14].

For many years, optimization of all-ceramic CFPDs is researched. Gabbert et al. [20] tested completely veneered zirconia CFPDs (12 mm^2^ connector cross section) replacing a premolar-sized molar and found mean fracture forces between 603 N and 703 N after aging for axially loads applied to the pontic. Reinforcement of the zirconia frameworks with an additional shoulder had no positive effect on the fracture loads. Fractures were usually located at the distal wall of the distal crown retainer, and no fractures of the connectors were observed. In a later study, Ohlmann et al. [21] tested zirconia CFPDs with similar configuration and different reinforcement modifications: Highest fracture loads were measured with zirconia frameworks reinforced at the oral wall of the distal abutment by a cervical shoulder (2.0 mm or 3.0 mm high, 1.0 mm wide), whereas a general thickening of the walls of the distal abutment (from 0.7 to 0.8 mm) or an isolated thickening of the occlusal surface (from 0.7 to 1.0 mm) resulted in only a slight increase in fracture load. Overall, with mean fracture loads ranging from 346 N to a maximum of 548 N, none of the tested groups achieved a load-bearing capacity justifying a recommendation for clinical use [21]. A further investigation dealing with additional zirconia framework reinforcements compared CFPDs differing in the wall thickness on the distal retainer crown [22]. With increased framework thickness of the distal crown (1 mm wall thickness) or application of an occlusal reinforcement (2 mm wide and 1 mm deep notch at the central fissure of the distal abutment tooth) corresponding mean fracture loads lay between 529 N and 590 N [22]. Regardless of the type of reinforcement, the predominant failure mode was still a partial breakout of the distal wall of the terminal abutment crown. The authors concluded that the fracture loads observed were not sufficient to recommend zirconia CFPDs without reservation for posterior tooth replacement [22]. With the current study, the monolithic design allocates all the space created by the tooth preparation to the zirconia framework, resulting in maximum reinforcement of the restorations and rather high fracture loads above 940 N (mean value 1181 N). This increased fracture resistance of the monolithic CFPD was to some extent expected and is consistent with previous mathematical estimates, including the fracture pattern in the area of the connection between the crown retainers [29]. Zhang et al. [29] used topology optimization and extended finite element method to propose an optimized design of posterior veneered zirconia CFPDs leading to higher fracture loads. For this purpose, they created a model of an all-porcelain CFPD that was iteratively modified by replacing porcelain elements with zirconia elements until crack initiation no longer occurred under a simulated vertical load of 250 N on the pontic and the abutment teeth. They found that especially reinforcement in the occlusal embrasures reduced the maximum principal stresses in porcelain ceramic CFPDs and that, when the occlusal surface was completely reinforced, the region of maximum tensile stress shifted to (i) the cervical embrasure of the connector between the abutment teeth and (ii) to the margin (mesial and distal) of the near-cantilever crown retainer [29]. What was initially surprising was the low fracture loads of the facially veneered CFPDs in our study compared to the monolithic CFPDs. Considering the common assumption that tensile forces occur mainly in the occlusal area when the pontic is loaded [25, 33], it was not expected that a facial veneer would reduce the fracture load of the restorations to such an extent (i.e., by more than half).

In this context, analysis of the fractured CFPDs using SEM provided further insights. In particular, the sharp edge of the veneering window proved to be very disadvantageous as it was found to be a site of predilection for fracture of the restorations in the area of the mesial connector, acting as a site of possible stress concentration [43]. In addition, material defects or fabrication or post-treatment defects were other origins of fractures of the ceramic CFPDs [26, 44] and, in the case of metal-ceramic restorations, deterioration of the ceramic veneer in the occlusal contact area [45].

As derived from 3D finite elements analysis, highest cantilever prostheses’ displacement and functional stresses can be produced (i) when a lateral loading direction of the pontic is chosen and (ii) when only the pontic is loaded [5]. Accordingly, oblique loading had an additional negative effect on the maximum load to failure of monolithic CFPDs in the present study. In this respect, the results of the monolithic CFPDs are consistent with those of another in vitro study of zirconia CFPDs, which showed that oblique loading of the pontic reduced the failure load by half compared to axial loading of the pontic [46]. For the monolithic CFPDs in the present study, this was accompanied by a change in failure mode from fracture in the mesial connector area to breakout of one or more crown walls. Interestingly, oblique loading did not reduce the fracture load of the partially veneered zirconia CFPDs, which could be due to the fact that the changed force vector moved the previous weak point (sharp-edged edge of the veneering window) out of the area of maximum tensile stress. From a clinical point of view, the results suggest that dynamic occlusion on cantilever pontics should be avoided through consistent functional occlusal design and occlusal adjustment measures.

Implant-supported CFPD are biomechanically a completely different system than CFPDs on resilient abutment teeth. As known from FEA, without periodontal resilience, the maximum principal stress can be expected at the top of the pontic connector just distal to the terminal abutment and the minimum principal stress at the bottom of the connector [33]. Accordingly, all but one of the implant-supported CFPDs failed in this region by fracture through the pontic connector.

With natural abutment teeth, no restoration failed, but the teeth fractured at rather low mean forces (392 N). Since extracted teeth are likely to be damaged during extracting, maximum loads with sound and vital teeth will likely be higher especially when considering clinical observations that tooth fractures can be expected in only about 3% of the CFPDs [47]. The results are also in contrast with the results of the study of Naumann et al. who tested a group of zirconia CFPDs on natural abutment teeth in a test setup very similar to that used in the present study [23]. Here, decementation rather than tooth fracture was the most common cause of failure, but mean failure loads (411 N) were in the same range as those found in the present study.

The use of artificial abutment teeth can have a tremendous effect on dental restorations. However, this is the case for thin-walled restorations and a load case where the fracture starts near the load application site [48]. For example, in minimally invasive zirconia crowns, the deflection magnitude of the thin occlusal zirconia layer will depend on the stiffness of the underlying structures, i.e., the fracture resistance will increase with increasing stiffness of the supporting structures (cement layer, enamel, dentin). For FDPs, which typically fracture far from the loading site, the abutment material is of minor importance and the deformation of the entire restoration, which is strongly influenced by the abutment resilience, is critical. This has been shown, for example, in FE analyses that complemented in vitro tests of inlay-retained FDPs [49]: in this publication, varying material parameters for abutment teeth and cement, as well as abutment tooth resilience, demonstrated that the simulation of tooth mobility in in vitro tests has a significant effect on fracture resistance. For all abutment materials except acrylic resin, no significantly different strains and stresses were found in the fracture-relevant connector areas compared to the situation with natural teeth. This will be even more true for the stresses and strains within the unsupported cantilever element of the CFPDs. Since tooth mobility was simulated, the fractures that occurred in the connector between the two abutment teeth were also not considerably influenced by the abutment tooth material. Therefore, it was assumed that the fracture forces for CFPDs found in this investigation should be in the same range as those found in a clinical setting.

It is a limitation of the study that zirconia CFPDs supported by implants or natural teeth were only investigated with a partially veneered design. However, no different results would be expected for monolithic CFPDs on natural teeth, since the failure of the restorations in the test was due to fracture of the abutment teeth. The case of implant-supported monolithic CFPDs might be different. Here, the complete omission of a veneer could also lead to an increase in fracture load. The “loading direction” factor was also not varied for all restoration groups, so no conclusions can be drawn about the fracture load of completely veneered zirconia CFPDs on CoCr tooth replicas and implant-supported CFPDs under oblique loading. For CFPDs on natural teeth, the previously made assumption that the teeth fail first in the test also appears to be valid for the variation of the load case. Another limitation is that, despite all standardization efforts, different connector diameters were used in the various groups. This does not refer to the difference between monolithic and completely veneered CFPDs. This is a logical consequence of the standardized external geometry, which led to veneered parts being replaced by monolithic zirconia. Rather, it concerns the partially veneered CFPDs on teeth and implants, whose different connection geometry to the supporting structure (tooth or implant) has slightly affected the connector area and thus influenced the absolute comparability of the results. Furthermore, two different veneering ceramics were used, one press ceramic and one layering ceramic. Both ceramics were recommended by the zirconia manufacturer and have a coefficient of thermal expansion matched to the zirconia material. With the press ceramic, the standardized geometry of the completely veneered CFPD could be implemented one-to-one by using a wax-milled space holder in the shape of the veneer. In contrast, the layering ceramic was used for the partially veneered CFPDs to vestibularly veneer them in a practice-oriented procedure. This may be a limitation of the study results in that full veneers are often layered by hand in daily practice, restricting the study findings for completely veneered CFPDs to overpressed restorations. This could be important, as it has been shown in the past that layered restorations have a higher fracture resistance than overpressed restorations when anatomically designed frameworks and comparable materials were used [50]. It is important to note that although the tested material is a zirconia with increased translucency, it is still a 3Y-TZP. It must therefore be distinguished from materials with increased yttria content. Future research approaches could therefore focus not only on design improvements of (partially) veneered zirconia CFPDs but also on material alternatives for the monolithic fabrication of zirconia CFPDs.

## Conclusions

Monolithic zirconia CFPDs may be considered a viable alternative to completely veneered metal-ceramic CFPDs with CoCr frameworks in terms of fracture load.

Oblique loading of the cantilever pontic drastically reduced the fracture load of monolithic zirconia CFPDs and should therefore be avoided in a clinical scenario.

Design flaws negatively affected the fracture load of the partially veneered zirconia CFPDs by promoting stress concentrations under axial loading due to the presence of sharp edges around the veneering windows.
